# Experimental data on load test and performance parameters of a LENZ type vertical axis wind turbine in open environment condition

**DOI:** 10.1016/j.dib.2017.10.071

**Published:** 2017-11-07

**Authors:** Seralathan Sivamani, Micha Premkumar T, Mohammed Sohail, Mohan T, Hariram V

**Affiliations:** Department of Mechanical Engineering, Hindustan Institute of Technology and Science, Hindustan University, Chennai, Tamil Nadu, India

**Keywords:** Vertical axis wind turbine, Lenz type, Performance, Two-stage, Open environment measurement

## Abstract

Performance and load testing data of a three bladed two stage LENZ type vertical axis wind turbine from the experiments conducted in an open environment condition at Hindustan Institute of Technology and Science, Chennai (location 23.2167°N, 72.6833°E) are presented here. Low-wind velocity ranging from 2 to 11 m/s is available everywhere irrespective of climatic seasons and this data provides the support to the researchers using numerical tool to validate and develop an enhanced Lenz type design. Raw data obtained during the measurements are processed and presented in the form so as to compare with other typical outputs. The data is measured at different wind speeds prevalent in the open field condition ranging from 3 m/s to 9 m/s.

**Specifications Table**

**Table:** 

Subject area	*Renewable energy*
More specific subject area	*Wind engineering*
Type of data	*Table, graphical figure*
How data was acquired	*Experimental study using vane anemometer, digital tachometer and dynamometer mechanism*
Data format	*Raw, filtered, calculated, tabulated, analyzed, plotted*
Experimental factors	*Data are normalized as per the standards used in wind turbine studies*
Experimental features	Vertical axis wind turbine working on the principle of combined lift and drag type is t*ested under natural wind conditions ranging from 3 m/s to 9 m/s*
Data source location	*Department of Mechanical Engineering, Hindustan Institute of Technology and Science, Padur 603 103, Tamil Nadu, India (*location 23.2167°N, 72.6833°E)
Data accessibility	*All the data are included in this article*

**Value of the data**

• The key focus of this data set on Lenz type VAWT is to provide technical information on this unique type of VAWT in order to quantify its aerodynamic behavior operating at an open field conditions.
• The presented data allows the researchers using numerical tool to validate and develop enhanced design based on this comprehensive full-scale measured data.
• The complete data set of this open field experimental study is conducted so as to provide a benchmark for future numerical analysis.

## 1 Data

**Fig. 1:**
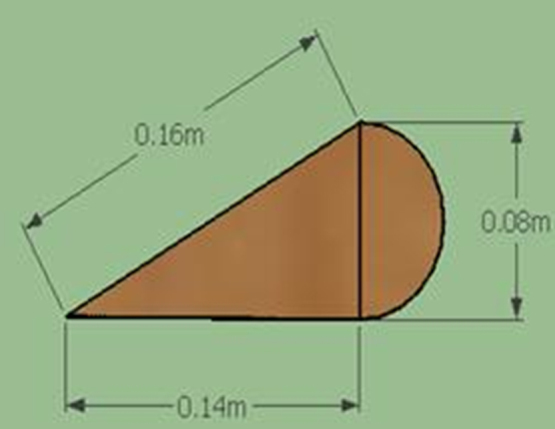
Dimensional detail of the Lenz type airfoil blade.

**Fig. 2:**
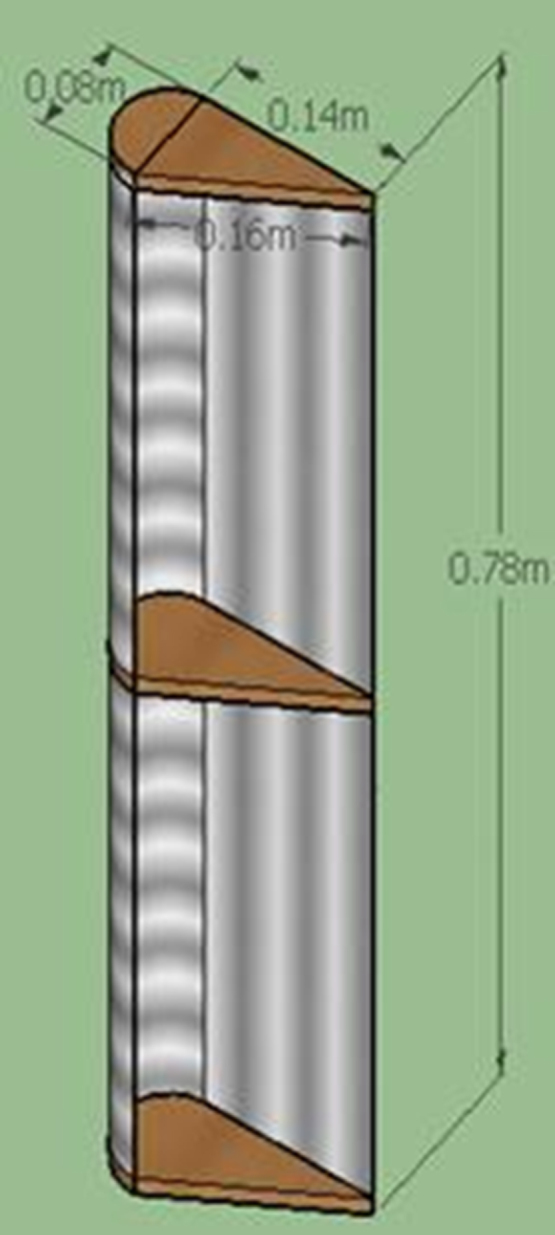
Schematic Lenz type blade.

**Fig. 3:**
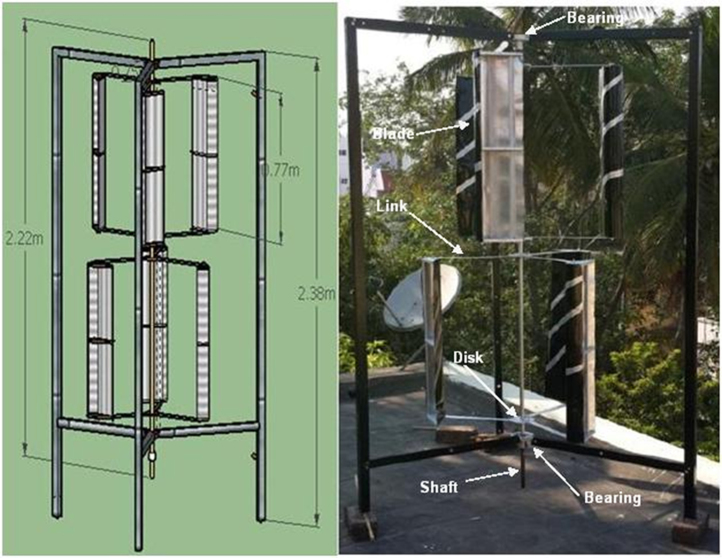
Schematic arrangement of the experimental Lenz type VAWT set up.

**Fig. 4:**
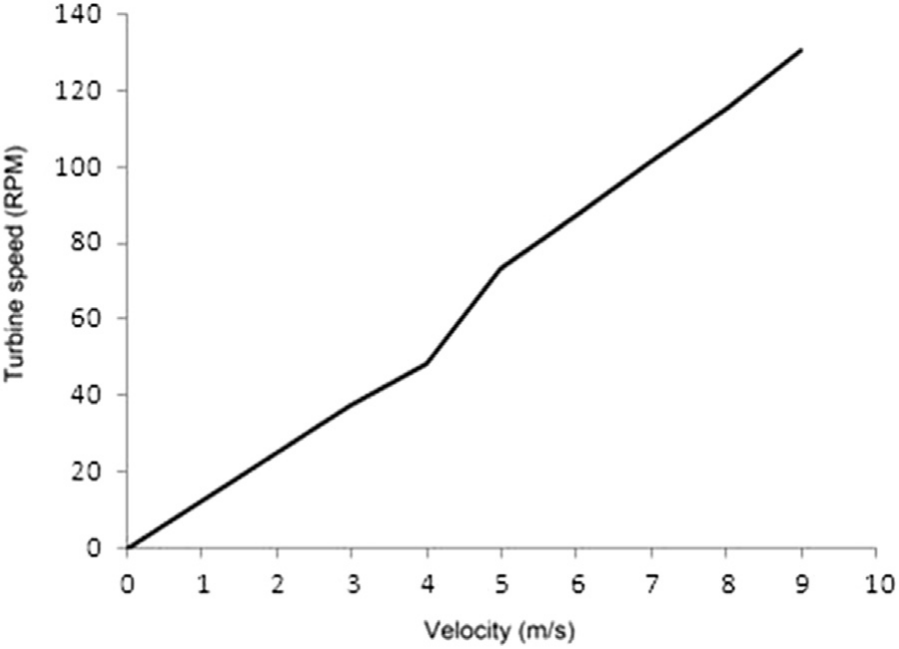
Wind turbine rotational speeds of Lenz type with wind velocity.

**Fig. 5:**
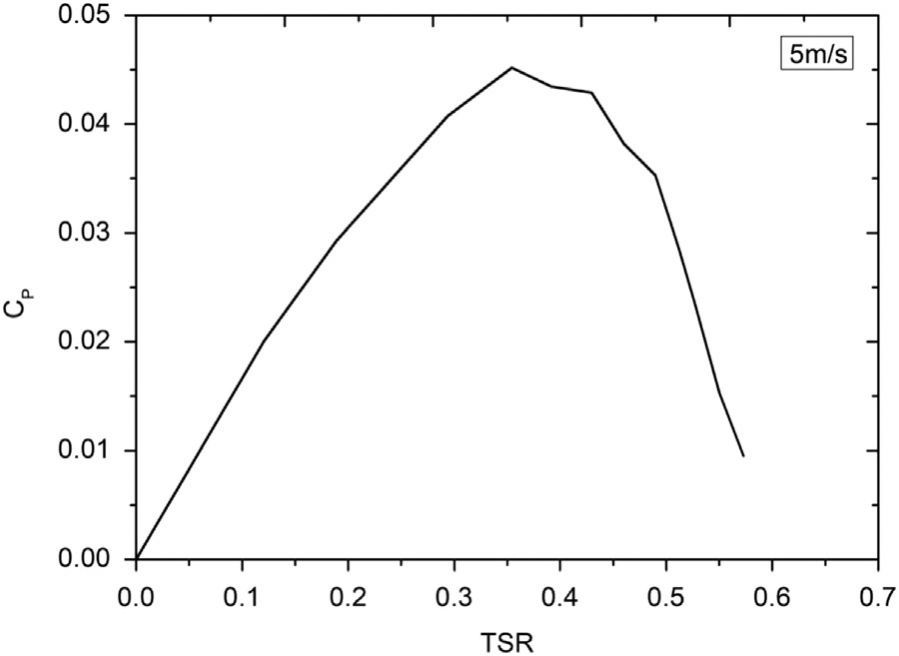
Variation of coefficient of power with tip speed ratio at wind velocity of 5 m/s.

**Fig. 6:**
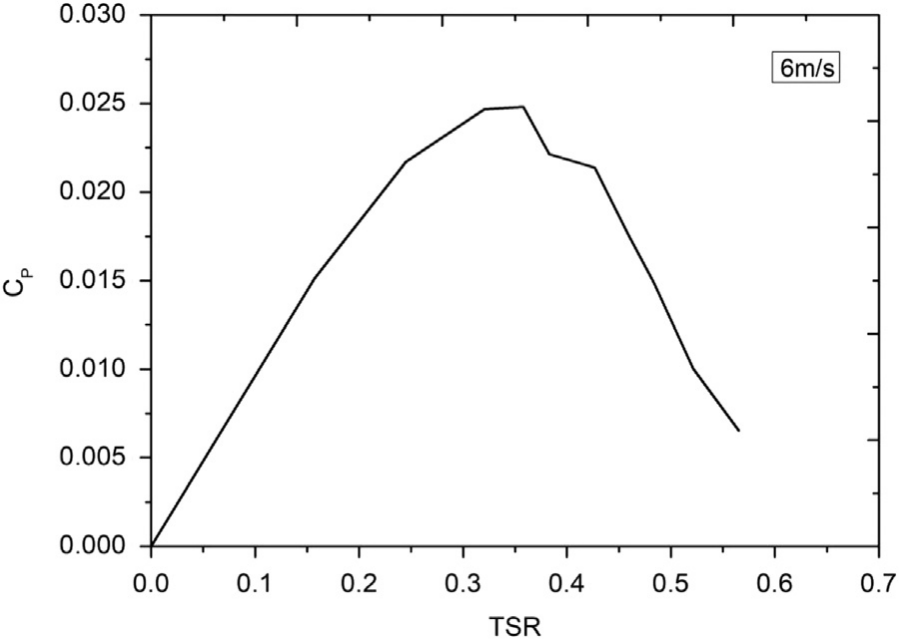
Variation of coefficient of power with tip speed ratio at wind velocity of 6 m/s.

**Fig. 7:**
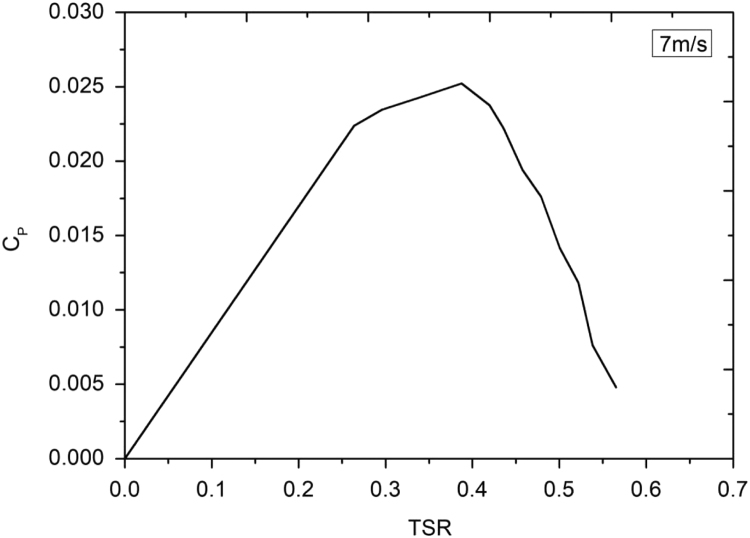
Variation of coefficient of power with tip speed ratio at wind velocity of 7 m/s.

**Fig. 8:**
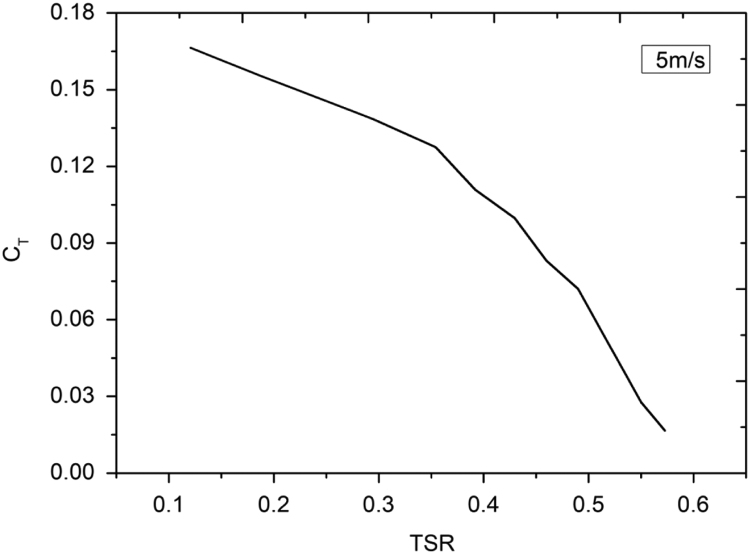
Variation of coefficient of torque with tip speed ratio at wind velocity of 5 m/s.

**Fig. 9:**
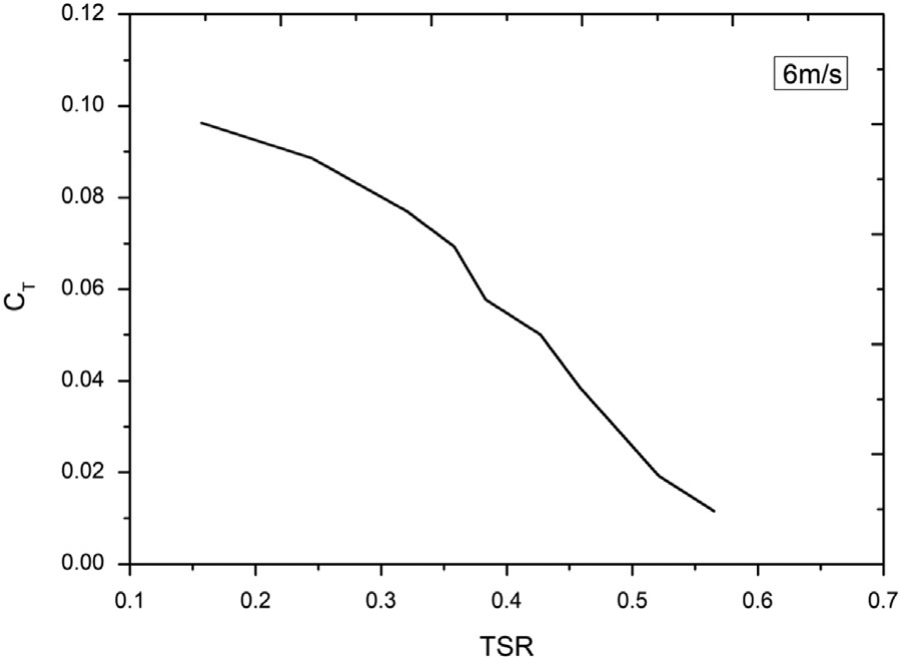
Variation of coefficient of torque with tip speed ratio at wind velocity of 6 m/s.

**Fig. 10:**
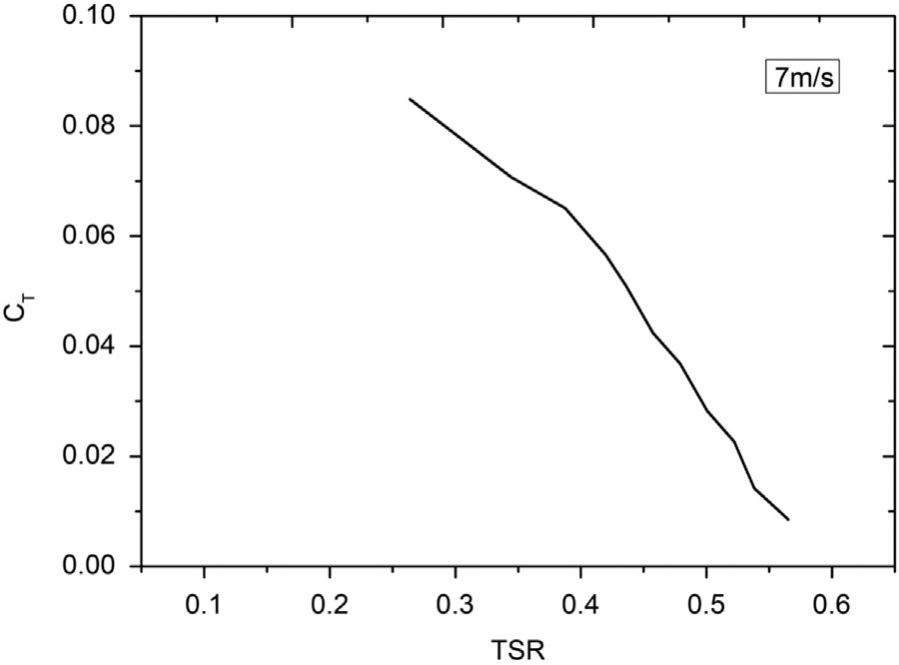
Variation of coefficient of torque with tip speed ratio at wind velocity of 7 m/s.

Lenz type vertical axis wind turbine [1], [2], [3], [4], as shown in Fig. 1, Fig. 2, Fig. 3 combines the working principle of lift and drag type vertical axis wind turbine (VAWT) and it is a suitable model for small scale power generation for domestic use. The design of this type of VAWT is developed by Dr. Edward Lenz. The raw data measured during the open environment test on Lenz type VAWT are wind speed (V), rotational speed of the rotor shaft and load applied on the dynamometer. These data are processed and presented as per the wind turbine research community approach. Data obtained are given in the Supplementary Table 1–4 which also provides the aerodynamics performance of the Lenz type VAWT in terms of dimensionless parameter as coefficient of power (C_p_) and coefficient of torque (C_T_). Further to disclose the influence of tip speed ratio (TSR) on aerodynamic performance, the variations of TSR with C_p_ and C_T_ at different wind velocities are shown in Fig. 4, Fig. 5, Fig. 6, Fig. 7, Fig. 8, Fig. 9, Fig. 10.

## 2 Experimental design, materials and methods

### 2.1 Wind turbine design and experimental setup

The Lenz type vertical axis wind turbine (VAWT) has the following components:

(a) Shaft: The shaft is made using solid mild steel material with diameter of 20 mm. The length of the shaft is 2220 mm.
(b) Blades: Lenz type airfoil blade profile is made using the material named “plywood” of thickness 12 mm. The dimensional detail of the Lenz type airfoil blade is given in Fig. 1. The span length of the each blade is 780 mm which is created by wrapping the airfoil with an aluminum sheet. Fig. 2 shows the schematic blade of the Lenz type used in the VAWT.
(c) Link: Steel link made of mild steel material is used to transfer the power from the blades to the shaft through the disk.
(d) Disk: Two disks made up of mild steel material of 150 mm diameter and 3 mm thick is used to transfer power from the links to the shaft and it is permanently fixed to the shaft.
(e) Pulley: A grooved pulley made of Teflon material is used to transfer the force exerted on the rope to the pulley. This force exerted on the rope act as the load applied on the VAWT.

Two self-lubricating heavy duty ball bearing with inner diameter of 20 mm is used to fix this Lenz type VAWT with the experimental test rig as seen in Fig. 3. Multi-staging helps in improving the self-starting capability of the VAWT [5], [6]. In this study, the Lenz type VAWT is designed as a two-stage arrangement with three blades in each stage which are placed 120° apart. The turbine in each stage is angularly offset by 60^0^ so as to allow the maximum amount of wind on the blades of VAWT from varying directions. Three blades arrangement is most preferred for the wind turbine rotor because two blades produces noise, rattle, imbalance and it is a poor self-starter at low-wind speeds. Fig. 3 shows the schematic arrangement of the experimental Lenz type VAWT set up. The specification details of the Lenz type VAWT is given in Table 1. Vane anemometer is used to measure the wind speed (V). Non-contact type digital tachometer is used to measure the rotational speed of the rotor shaft (N). Loading on the Lenz type VAWT is carried out by using a brake drum dynamometer which is used to measure the torque (T). The pulley, spring balance and weighing pan are connected using 1 mm fishing nylon type wire string. The measurement of torque in the Lenz type VAWT could be affected by the friction in the nylon wire string wound around the pulley on the rotor shaft and bearings. Necessary care is taken to reduce the friction by washing the bearings in petrol in order to remove the presence of grease. The experiments are carried out in an open atmosphere at Hindustan Institute of Technology and Science, Padur, near Chennai (location 23.2167°N, 72.6833°E) with wind velocity varying from 3 m/s to 9 m/s. The Lenz type VAWT is tested from no load to maximum condition by applying loads in the dynamometer.

**Table 1:** Specification details of the Lenz type VAWT.

Shape of the blade = Lenz type
Aspect ratio (AR = H/D)= 1.85
Diameter of the rotor (D) = 840 mm
Height of the rotor (H) = 1560 mm
No. of stage = 2
No. of blade in each stage = 3

### 2.2 Performance and loading test data

The performance indices of the Lenz type VAWT is measured in terms of power coefficient (C_p_) and torque Coefficient (C_T_). In this present study, the airstream is flowing through a swept area or frontal area (A) and the mass flow rate is given as ρAV. The power available in the air is given by

$$P_{available}=Kinetic\;energy\times mass\;flow\;rate$$

$$\begin{array}{c} P_{available}=\left(\frac{V^{2}}{2}\right)\rho AV=\frac{\rho AV^{3}}{2} \end{array}$$

The rotor shaft power is found from the rotational speed of the rotor (N) and brake torque produced (T) and it is given as

$$P_{shaft}=\frac{2\pi NT}{60}=\frac{2\pi N\left(F\times r_{p}\right)}{60}$$

The ratio of shaft power or actual power produced by the turbine (P_shaft_) to the power available in the wind (P_available_) is known as power coefficient (C_p_) which indicates the efficiency of conversion.

Torque coefficient (C_T_) is given as $C_{T}=\frac{T}{\frac{1}{2}\rho AV^{2}R}=\frac{F\times r_{p}}{\frac{1}{2}\rho AV^{2}R}$

where V is the free stream wind speed (m/s), r_p_ is the radius of the pulley attached to the shaft (m), F is the mechanical load applied to the turbine shaft (N), R is the rotational radius of the turbine (m), N is the rotational speed (rpm) and T is the torque applied on the turbine (Nm).

These performance indices are evaluated with respect to tip speed ratio (λ) which is given by

$$\lambda =\frac{u}{V}=\frac{\omega R}{V}=\frac{2\pi N}{60}\frac{R}{V}$$

where ω is angular speed (rad/s) and *u* is the blade tip speed (m/s).

As the present data is obtained by testing the Lenz type VAWT under open field environmental conditions, a stable wind flow could not be obtained. Therefore, the experiment is repeated in different periods and days to remove any uncertainty in the measurements and data. Mechanical load is applied in a gradual manner on the rotor shaft of the VAWT. At a given load condition, the rotor is loaded for different wind velocities prevalent in the open field conditions and the values of the load and spring balance readings are recorded to calculate power coefficient (C_p_) and torque Coefficient (C_T_) with respect to tip speed ratio (λ).

As can be seen in Fig. 4, initially the rotor shaft of the Lenz type VAWT is in stationary condition. At different wind speed prevalent in the open field conditions, ranging from 3 m/s to 9 m/s, the rotational speed of the rotor shaft is found using digital tachometer (Supplementary Table 1). As can be observed from the data, increase in wind velocity improved the rotational speed of the rotor shaft and a maximum turbine speed of 131 RPM is attained at wind velocity of 9 m/s.

As the load on the VAWT is increased, the rotational speed of the Lenz type VAWT is reduced as can be seen in the data shown in Supplementary Table 2–4. As can be seen in Fig. 5, Fig. 6, Fig. 7, at wind velocity 5 m/s, the C_pmax_ of 0.045175 at λ = 0.354192 is obtained. Similarly, C_pmax_ of 0.024813 at λ = 0.35796 and C_pmax_ of 0.02522 at λ = 0.387566 is obtained for wind velocity 6 m/s and 7 m/s respectively. As seen in Fig. 8, Fig. 9, Fig. 10, it is observed that the torque coefficient values decreases with increase in the tip speed ratios. This is due to gradual increase in the load applied on the rotor shaft which led to the reduction in VAWT rotational speed. The C_p_ value rises with increase in tip speed ratio up to certain maximum value and later, it droops for further increase in the tip speed ratio.
