# Supplementary material for: Experimental data on load test and performance parameters of a LENZ type vertical axis wind turbine in open environment condition
Source: Data Brief. 2017 Nov 7;15:1035–42. doi: 10.1016/j.dib.2017.10.071 (PMC5686457; doi:10.1016/j.dib.2017.10.071)
Supplement: Supplementary file 1 — Supplementary material [file mmc1.docx]

**Disclosure statement**

The authors state that there is no potential conflict of interest in this data. Also, this research work is not supported by any funding agencies and this is solely carried out by the authors.
