# Supplementary material for: Experimental data on load test and performance parameters of a LENZ type vertical axis wind turbine in open environment condition
Source: Data Brief. 2017 Nov 7;15:1035–42. doi: 10.1016/j.dib.2017.10.071 (PMC5686457; doi:10.1016/j.dib.2017.10.071)
Supplement: Supplementary file 2 — Supplementary material [file mmc2.docx]

**APPENDIX A: Supplementary Data**

**Table 1** Data of wind turbine rotational speed with wind velocity

| **Velocity, V (m/s)** | **Rotational speed, N (rpm)** |
| --- | --- |
| 3 | 38 |
| 4 | 49 |
| 5 | 74 |
| 6 | 88 |
| 7 | 102 |
| 8 | 116 |
| 9 | 131 |

**Table 2** Performance parameters at wind velocity of 5 m/s

| **Velocity, V (m/s)** | **Rotational speed, N (rpm)** | **Torque, T (N/m)** | **Tip Speed Ratio, λ** | **Coefficient of**  **Torque, C_T_** | **Coefficient of**  **power, C_p_** |
| --- | --- | --- | --- | --- | --- |
| 5 | 76 | 0.103005 | 0.572736 | 0.016636 | 0.009528 |
| 5 | 73 | 0.171675 | 0.550128 | 0.027727 | 0.015253 |
| 5 | 70 | 0.27468 | 0.52752 | 0.044363 | 0.023402 |
| 5 | 68 | 0.34335 | 0.512448 | 0.055454 | 0.028417 |
| 5 | 65 | 0.446355 | 0.48984 | 0.07209 | 0.035313 |
| 5 | 61 | 0.515025 | 0.459696 | 0.083181 | 0.038238 |
| 5 | 57 | 0.61803 | 0.429552 | 0.099817 | 0.042877 |
| 5 | 52 | 0.6867 | 0.391872 | 0.110908 | 0.043462 |
| 5 | 47 | 0.789705 | 0.354192 | 0.127544 | 0.045175 |
| 5 | 39 | 0.858375 | 0.293904 | 0.138635 | 0.040745 |
| 5 | 25 | 0.96138 | 0.1884 | 0.155271 | 0.029253 |
| 5 | 16 | 1.03005 | 0.120576 | 0.166361 | 0.020059 |

**Table 3** Performance parameters at wind velocity of 6 m/s

| **Velocity, V (m/s)** | **Rotational speed, N (rpm)** | **Torque, T (N/m)** | **Tip Speed Ratio, λ** | **Coefficient of**  **Torque, C_T_** | **Coefficient of**  **power, C_p_** |
| --- | --- | --- | --- | --- | --- |
| 6 | 90 | 0.103005 | 0.5652 | 0.011553 | 0.00653 |
| 6 | 83 | 0.171675 | 0.52124 | 0.019255 | 0.010036 |
| 6 | 77 | 0.27468 | 0.48356 | 0.030808 | 0.014897 |
| 6 | 73 | 0.34335 | 0.45844 | 0.03851 | 0.017654 |
| 6 | 68 | 0.446355 | 0.42704 | 0.050062 | 0.021379 |
| 6 | 61 | 0.515025 | 0.38308 | 0.057764 | 0.022128 |
| 6 | 57 | 0.61803 | 0.35796 | 0.069317 | 0.024813 |
| 6 | 51 | 0.6867 | 0.32028 | 0.077019 | 0.024668 |
| 6 | 39 | 0.789705 | 0.24492 | 0.088572 | 0.021693 |
| 6 | 25 | 0.858375 | 0.157 | 0.096274 | 0.015115 |

**Table 4** Performance parameters at wind velocity of 7 m/s

| **Velocity, V (m/s)** | **Rotational speed, N (rpm)** | **Torque, T (N/m)** | **Tip Speed Ratio, λ** | **Coefficient of**  **Torque, C_T_** | **Coefficient of**  **power, C_p_** |
| --- | --- | --- | --- | --- | --- |
| 7 | 105 | 0.103005 | 0.5652 | 0.008488 | 0.004797 |
| 7 | 100 | 0.171675 | 0.538286 | 0.014146 | 0.007615 |
| 7 | 97 | 0.27468 | 0.522137 | 0.022634 | 0.011818 |
| 7 | 93 | 0.34335 | 0.500606 | 0.028293 | 0.014164 |
| 7 | 89 | 0.446355 | 0.479074 | 0.036781 | 0.017621 |
| 7 | 85 | 0.515025 | 0.457543 | 0.042439 | 0.019418 |
| 7 | 81 | 0.61803 | 0.436011 | 0.050927 | 0.022205 |
| 7 | 78 | 0.6867 | 0.419863 | 0.056586 | 0.023758 |
| 7 | 72 | 0.789705 | 0.387566 | 0.065073 | 0.02522 |
| 7 | 64 | 0.858375 | 0.344503 | 0.070732 | 0.024367 |
| 7 | 55 | 0.96138 | 0.296057 | 0.07922 | 0.023454 |
| 7 | 49 | 1.03005 | 0.26376 | 0.084878 | 0.022387 |
